# Characterization of Erythromycin and Tetracycline Resistance in *Lactobacillus fermentum* Strains

**DOI:** 10.1155/2018/3912326

**Published:** 2018-11-11

**Authors:** Elizaveta Anisimova, Dina Yarullina

**Affiliations:** Department of Microbiology, Kazan Federal University, Kremlevskaya Str. 18, Kazan 420008, Russia

## Abstract

*Lactobacillus fermentum* colonizing gastrointestinal and urogenital tracts of humans and animals is widely used in manufacturing of fermented products and as probiotics. These bacteria may function as vehicles of antibiotic resistance genes, which can be transferred to pathogenic bacteria. Therefore, monitoring and control of transmissible antibiotic resistance determinants in these microorganisms is necessary to approve their safety status. The aim of this study was to characterize erythromycin and tetracycline resistance of *L. fermentum* isolates and to estimate the potential transfer of resistance genes from lactobacilli to the other Gram-positive and Gram-negative bacteria. Among six *L. fermentum* strains isolated from human feces and commercial dairy products, five strains demonstrated phenotypic resistance to tetracycline. PCR screening for antibiotic resistance determinants revealed plasmid-located tetracycline resistance genes *tet*(K) and *tet*(M) in all strains and erythromycin resistance genes *erm*(B) in the chromosome of *L. fermentum* 5-1 and *erm*(C) in the plasmid of *L. fermentum* 3-4. All tested lactobacilli lacked conjugative transposon Tn*916* and were not able to transfer tetracycline resistance genes to *Staphylococcus aureus*, *Staphylococcus epidermidis*, *Listeria monocytogenes*, *Acinetobacter baumannii*, *Citrobacter freundii*, and *Escherichia coli* by filter mating. *Staphylococcus haemolyticus* did not accept erythromycin resistance genes from corresponding *Lactobacillus* strains. Thus, in the present study, *L. fermentum* was not implicated in the spread of erythromycin and tetracycline resistance, but still these strains pose the threat to the environment and human health because they harbored erythromycin and tetracycline resistance genes in their plasmids and therefore should not be used in foods and probiotics.

## 1. Introduction

*Lactobacillus fermentum* is a common inhabitant of human gastrointestinal and urogenital tracts where these bacteria are known to exert health-promoting effects [1, 2]. It is naturally present in raw milk, dairy products, and many traditional fermented foods and beverages [3, 4]. Some probiotic isolates, such as *L. fermentum* RC-14 and *L. fermentum* 90 TC-4, are already widely used and produced on an industrial scale [5, 6]. The species is listed in the qualified presumption of safety (QPS) published by the European Food Safety Authority (EFSA) [7], and along with other lactic acid bacteria (LAB), it has been generally recognized as safe (GRAS status) by the U.S. Food and Drug Administration. However, over the last decade, bacteria used as probiotics or in starter cultures have received growing attention with regard to their potential involvement in acquisition and transfer of antibiotic resistance genes to food or gut pathogens [8, 9].

In line with the growing awareness of LAB involvement in the spread of antibiotic resistance, it is imperative that the antibiotic resistance profile of probiotic and starter cultures should be studied both at the physiological level and molecular level. A phenotypically resistant strain may be genotypically “susceptible,” and in contrast, a susceptible phenotype may carry silent genes, which are observed with genotyping [10]. Determination of antibiotic resistance among lactobacilli is confounded by the absence of standards for susceptibility testing. Microbiological breakpoints have been determined only for some antibiotics and for particular most commonly used probiotic species [11]. Besides, minimum inhibitory concentration (MIC) breakpoint values can vary depending on the method and media applied, as well as between species of the same genus [12–14].

Bacterial resistance to antibiotics can be of two types: intrinsic resistance, which is a natural property of an organism, having a minimal potential for horizontal spread and posing no risk in nonpathogenic bacteria, and acquired resistance, which results from mutation or, what is more often, via the acquisition of generic materials, such as a plasmid or a transposon [10]. Lactobacilli have been reported to have a high natural resistance to vancomycin, aminoglycosides, and the majority of nucleic acid inhibitors [15]. Resistance to other antibiotics varies greatly among species. Erythromycin resistance and tetracycline resistance have been found to be acquired, and therefore, corresponding genetic determinants are often considered potentially transferable [16, 17]. Acquired resistance to erythromycin encoded by the *erm*(B) gene has been detected in two *L. fermentum* strains NWL24 and NWL26 isolated from Chinese fermented foods (yogurt) [18]. *L. fermentum* CB101 also isolated from Chinese fermented food (cucumbers) showed resistance to both erythromycin and tetracycline with very high MICs (512 *μ*g/ml for erythromycin and more than 128 *μ*g/ml for tetracycline) [19]. From four erythromycin- and tetracycline-resistant *L. fermentum* strains isolated from Indian fermented foods, three strains have been found to harbor the *erm*(B) gene and one harbors the tetracycline efflux genes *tet*(K) and *tet*(L) [20]. Several plasmids potentially implemented in transfer of antibiotic resistance have been identified in *L. fermentum*, e.g., resistance plasmid pLME300 of *L. fermentum* ROT1 [21], tetracycline and erythromycin resistance plasmids of *L. fermentum* LF601 [22], and plasmid pLEM3 conferring erythromycin resistance of *L. fermentum* [23]. The works demonstrating conjugative transfer of antibiotic resistance determinants from lactobacilli to other bacteria especially those belonging to the gut microbiota are very limited [24–26]. So far, there is only one evidence of successful transfer of the *erm*(B) gene from *L. fermentum* NWL24 to *Enterococcus faecalis* 181 in filter mating experiments [18].

The aim of this work was to comprehensively characterize erythromycin and tetracycline resistance profiles of *L. fermentum* strains and evaluate the transferability of corresponding resistance genes to other bacteria. In this study, six *L. fermentum* strains isolated from human feces and commercial dairy products were assayed for susceptibility to erythromycin and tetracycline by the agar disc diffusion method, followed by determination of MICs for these antibiotics by the broth microdilution method. Phenotypic assays of bacterial strains were complemented by direct screening for the presence of erythromycin and tetracycline resistance determinants by PCR amplification. The transferability of these antibiotic resistance genes from *L. fermentum* strains to a number of Gram-positive and Gram-negative bacteria was studied in filter mating experiments. This study provides a reference for the safety assessment and contributes to the evaluation system of probiotics.

## 2. Materials and Methods

### 2.1. Isolation of Bacteria and Growth Conditions

Six *Lactobacillus* strains were isolated from the feces of healthy individuals (HF-A1, HF-A4, and HF-B1) and commercial dairy products (3-4, 5-1, and 5-2) by serial dilution of the sample in sterile phosphate-buffered saline (PBS), subsequent plating onto the de Man–Rogosa–Sharpe (MRS) agar (HiMedia, India), and incubation under anaerobic conditions (Anaerogas pack; NIKI MLT, Russia) at 37°C. Single colonies were selected, purified, and maintained on the MRS agar for immediate use and in 20% glycerol for storage at −80°C. Prior to all experiments, bacterial isolates were subcultured at least twice.

### 2.2. Identification of Bacteria

The bacterial colonies were assigned to the genus *Lactobacillus* by MALDI-TOF mass spectrometry (Bruker Biotyper system, Bruker Daltonics, Germany), as previously described [27]. In brief, a single fresh colony from the MRS agar was smeared onto a ground steel target (Bruker Daltonik), overlaid with 1 *μ*l of a saturated solution of alpha-cyano-4-hydroxycinnamic acid matrix in 50% acetonitrile and 2.5% trifluoroacetic acid, and air-dried at room temperature. For each strain, two preparations of the colony material were analyzed. Mass spectra were recorded according to the manufacturer's instructions. The obtained spectra were compared with reference spectra in the integrated database (version 3.2.1.1). Standard Bruker interpretative criteria were applied. Scores ≥2.3 were accepted for reliable species assignment and scores ≥2.0 but <2.3 for reliable identification to the genus level. Scores below 2.0 were considered unreliable.

For most accurate species identification, the 16S rRNA gene was amplified by the PCR method using universal 16S rRNA bacterial primers 27F and 1392R (Table 1) and PCR program described below. The PCR-amplified 1.4 kbp DNA fragments were resolved by agarose gel electrophoresis, purified, and sequenced on an ABI Prism 3730 sequencer (Applied Biosystems). The bacterial isolates were identified to the species level by comparing their 16S rRNA gene sequences with those in the NCBI databases by BLAST.

### 2.3. Antibiotic Susceptibility Testing and MIC Determination

Susceptibility to erythromycin and tetracycline was determined by the disc diffusion method, as described earlier [27]. In brief, bacteria were grown in the MRS broth overnight at 37°C in anaerobic conditions (Anaerogas pack; NIKI MLT, Russia) and pour-plated on MRS agar plates (0.5 McFarland after inoculation). Antibiotic discs (Scientific Research Centre of Pharmacotherapy, Russia) were placed on the surface of inoculated plates. After 48 h incubation in anaerobic conditions at 37°C, the diameter of the inhibition zone was measured and interpreted as susceptible (S), moderately susceptible (MS), or resistant (R) according to the method in [27]. The following standards for interpreting the zones of inhibition for antibiotics were used: bacteria were considered resistant (R) to erythromycin (15 *μ*g/disc) when the zone of inhibition (mm) was ≤10, moderately susceptible (MS) at 11–20, and susceptible (S) at ≥21; bacteria were considered resistant (R) to tetracycline (30 *μ*g/disc) when the zone of inhibition (mm) was ≤16, moderately susceptible (MS) at 17–21, and susceptible (S) at ≥22.

The MIC for antibiotics was determined by the broth microdilution method in the MRS broth in 96-well nontreated cell culture plates (Eppendorf). Tetracycline (Sigma-Aldrich) and erythromycin (Sigma-Aldrich) were tested in the concentration range of 0.12–256 *μ*g/ml obtained after a series of twofold dilutions in the MRS broth. Wells were inoculated with 200 *μ*l of the bacterial culture (3 × 10^7^ CFU/ml) and incubated at 37°C. The MIC was read after 48 h of incubation as the lowest concentration of an antibiotic at which visible growth was inhibited. According to the microbiological breakpoints suggested for *L. fermentum* by EFSA [11], strains with MICs higher than 1 *μ*g/ml for erythromycin and 8 *μ*g/ml for tetracycline were considered resistant.

### 2.4. DNA Preparation and Manipulations

Bacteria were cultivated overnight at 37°C in 30 ml of the MRS broth. The cells were harvested by centrifugation (5 min, 13 000 RPM) and then resuspended in 3 ml of the lysis buffer (50 mM Tris-HCl and 3 mg/ml lysozyme, pH 8.0). The resuspended pellet was incubated 3 h at 37°C with shaking. After this step, 100 *μ*l of sodium dodecyl sulfate (SDS; 10%, w/v) wad added, and the sample was gently inverted for cell disruption. 500 *μ*l of phenol : chloroform (1 : 1, v/v) was added. After a centrifugation step (5 min, 13 000 RPM, room temperature), the clear supernatant was transferred to a new tube. The DNA was precipitated with 500 *μ*l of cold isopropanol, and the DNA was taken out with a plastic tip. The DNA was washed with ethanol 96% (w/v), and after it has been dried, it was diluted in 100 *μ*l of distilled water. All the DNA samples were stored at −20°C.

Plasmid DNA was obtained using GeneJET Plasmid Miniprep Kit (Thermo Scientific), according to the manufacturer's instructions.

### 2.5. PCR Detection of Genes

The presence of erythromycin and tetracycline resistance genes was determined by PCR amplification using the primers listed in Table 1. The presence of the transposon Tn*916* was detected by amplification of the integrase-encoding gene (*Int*) and examining the interregion between *tet*(M) and *Int* genes with the primer *Tet-int* described in Table 1.

The PCR reaction was carried out in a total volume of 25 *μ*l containing DNA template, 10 pmol of each primer (Table 1), 1 U Taq DNA polymerase, each of four dNTPs at a concentration of 200 *μ*M, and PCR buffer containing Tris-HCl, KCl, (NH_4_)_2_SO_4_, MgSO_4_, and Triton X-100. The amplification program was as follows: initial denaturation at 95°C for 4 min, 35 cycles at 94°C for 30 s, 46–60°C (according to the annealing temperature for the individual primers; Table 1) for 30 s, and 72°C for 1.5 min, and a final extension step at 72°C for 7 min. Positive and negative controls from our lab were used for all PCR reactions. PCR products (5 *μ*l) were separated by electrophoresis on a 1% agarose gel, which was stained with Midori Green DNA Stain (Nippon Genetics Europe, Germany).

### 2.6. Filter Mating Experiments

Transferability of antibiotic resistance from lactobacilli was examined by filter mating, as described in [24], on a number of Gram-positive (*Staphylococcus aureus* subsp. *aureus* ATCC® 29213™, *Staphylococcus haemolyticus*, *Staphylococcus epidermidis* (clinical isolates), and *Listeria monocytogenes* 88K (a gift from Dr. Alexey Vasilchenko, Orenburg State University)) and Gram-negative (*Acinetobacter baumannii*, *Citrobacter freundii*, and *Escherichia coli* (clinical isolates)) bacteria. Clinical isolates of *S. haemolyticus*, *S. epidermidis*, and *E. coli* were obtained from the Kazan Institute of Epidemiology and Microbiology (Kazan, Russia). Clinical isolates of *A. baumannii* and *C. freundii* were obtained from the Institute of Medical Microbiology (Giessen, Germany). Cultures of pathogens were incubated in the Luria-Bertani (LB) medium. Antibiotic resistance profiles of recipient strains were characterized by determination of MICs, as described above. The lack of erythromycin and tetracycline resistance genes was proved by PCR, as indicated earlier. *S. haemolyticus* was sensitive to both erythromycin and tetracycline but carried the *tet*(K) gene and therefore was used as the recipient strain for erythromycin resistance genes. Other tested strains were either resistant to erythromycin (all Gram-negative strains) or possessed a silent *erm*(B) gene (*S. aureus*, *S. epidermidis*, and *L. monocytogenes*). As they were sensitive to tetracycline and lacked *tet* genes, they were used as recipient strains for tetracycline resistance genes. Chloramphenicol (40 *μ*g/ml) was used as a selective antibiotic marker for transconjugants of *S. epidermidis*, *S. haemolyticus*, and *A. baumannii*, rifampicin (2.5 *μ*g/ml) for *S. aureus*, *L. monocytogenes*, and *E. coli*, and ampicillin (40 *μ*g/ml) for *C. freundii*. These concentrations were sufficient to completely inhibit growth of *Lactobacillus*, whereas recipient bacteria were unaffected.

For filter mating experiments, the MRS broth and LB broth were inoculated (1 : 100) with overnight grown donor *L. fermentum* cultures and recipient pathogen cultures, correspondingly, and incubated at 37°C for approx. 4 h to the mid-exponential phase of growth. One ml of each culture was mixed and filtered through a sterile 0.45 *μ*m pore-size nitrocellulose membrane filter (Millipore, USA). After that, sterile peptone physiological saline (PPS) solution (8.5 g/l NaCl and 1 g/l bacteriological peptone) was passed through the filter to trap the cells more tightly into the membrane. The filters were incubated overnight on the LB agar at 37°C. The bacteria were washed from the filters with 2 ml PPS. Dilutions of the mating mixtures were spread onto LB agar plates containing 10 *μ*g/ml tetracycline or erythromycin (Sigma-Aldrich) and selective antibiotic (chloramphenicol, rifampicin, or ampicillin, all from Sigma-Aldrich) (double selective medium), agar plates containing 10 *μ*g/ml tetracycline or erythromycin (Sigma-Aldrich) or selective antibiotic (single selective medium), and nonselective medium without antibiotics. Control cultures of donor and recipient strains were also individually plated on three types of agar plates. Plates were incubated at 37°C for 24–48 h.

## 3. Results and Discussion

### 3.1. Isolation and Identification of *L. fermentum* Strains

In our previous work, among 15 *Lactobacillus* strains isolated from feces of healthy volunteers (unpublished data) and 19 *Lactobacillus* strains isolated from commercial dairy products and probiotics [27], six strains were shown to be resistant to either erythromycin or tetracycline by the disc diffusion method. Resistance to these two antibiotics is considered the most threatening one in probiotics because it is often acquired and potentially transferable [16, 17]. Therefore, these six *Lactobacillus* strains were used in this study to comprehensively characterize their erythromycin or tetracycline resistance profiles and evaluate the transferability of corresponding resistance genes to other bacteria. All tested strains were putatively assigned to *L. fermentum* species by MALDI Biotyper as sample mass spectra shared the maximum similarity with the reference mass spectrum of *L. fermentum* from MALDI Biotyper software (score values ranged between 2.019 and 2.083). This finding was verified by 16S rRNA gene analysis after we determined that sample 16S rDNA sequences had a similarity score of ≥99% with that of reference sequences in the NCBI database classified as *L. fermentum*.

### 3.2. Phenotypic Profile of Antimicrobial Resistance

Phenotypic profile of *L. fermentum* resistance to erythromycin and tetracycline is presented in Table 2. All strains tested in this study were susceptible or moderately susceptible to erythromycin by disc diffusion, except for the strain 5-1, which was resistant. With MICs between 0.25 and 1 *μ*g/ml, all strains were considered susceptible to erythromycin based on the MIC breakpoints established by EFSA [11]. Five tested *L. fermentum* isolates showed resistance to tetracycline, whereas the strain 5-1 was moderately susceptible. In full concordance with the results of the disc diffusion method, these five isolates of *L. fermentum* showed high MICs of 16–64 *μ*g/ml for tetracycline and thus were assigned as resistant to tetracycline according to [11]. Tetracycline resistance common among the human fecal isolates may be explained by the intensive use of tetracyclines, alone or in combination, in medicine for prophylaxis or therapy and in consumed food, which could provide the selective pressure needed for antibiotic-resistant bacteria to develop and spread. The phenotypic profile of antimicrobial resistance characterized by disc diffusion mainly coincided with observed MICs, except for the strain *L. fermentum* 5-1, which was resistant to erythromycin in the disc diffusion method, but ultimately was considered nonresistant according to the MIC value.

Thus, phenotypic assays showed that *L. fermentum* strains HF-A1, HF-A4, HF-B1, 3-4, and 5-2 possess tetracycline resistance which is potentially transferable.

### 3.3. Antibiotic Resistance Genes

Antibiotic resistance genes were detected by PCR, and the results are presented in Table 2. All the strains were tested for the presence of erythromycin resistance genes *erm*(A), *erm*(B), *erm*(C), *erm*(T), and *mef*(A). Only *L. fermentum* 5-1 was positive for the *erm*(B) gene, giving a 425 bp band, corresponding to its resistance phenotype in the disc diffusion assay (Figure S1B, lane 2). The *erm*(B) gene, which encodes an rRNA methylase acting on the 23S ribosomal subunit, is the most frequently found one of all erythromycin resistance genes in *Lactobacillus* species [15]. According to PCR results, the *erm*(B) gene was attributed to chromosomal DNA in *L. fermentum* 5-1 and thus is not likely to be transferable. However, acquired resistance to erythromycin because of *erm*(B) has often been reported in lactobacilli from different sources [9, 18, 20, 21, 23, 35]. The erythromycin resistance gene *erm*(C) was found in plasmid DNA from *L. fermentum* 3-4 (Figure S1B, lane 3), and to our knowledge, this gene has not been described previously from that particular species. Its lower prevalence among lactobacilli is consistent with previous reports [20, 35]. The *erm*(C) gene has only been detected in few *L. plantarum* strains from fermented dry sausages [36] and several chicken isolates of the species *L. salivarius*, *L. agilis*, *L. crispatus*, *L. reuteri*, and *L. saerimneri* [37]. Other erythromycin resistance determinants (*erm*(T) and *mef*(A)) were not detected in any strain.

In this study, we also tested the presence of tetracycline resistance genes *tet*(K), *tet*(M), *tet*(W), *tet*(S), and *tet*(L). The *tet*(K) and *tet*(M) genes were found in plasmid DNA of all the tested strains (Figures S1C and S1D). These determinants have different modes of action: *tet*(K) encodes efflux pumps, whereas *tet*(M) offers ribosomal protection [16]. The simultaneous occurrence of *tet*(K) and *tet*(M) genes is in agreement with reports of other lactobacilli containing multiple *tet* genes [18, 20, 37–39]. Genetic location of the *tet*(K) gene on small multicopy plasmids and *tet*(M) on conjugative transposons (Tn*916*-Tn*1545* family) promotes the spread of these determinants [16]. Yet in this study, we have shown that all the tested *L. fermentum* strains lack the conjugative transposon Tn*916*, carrying the *tet*(M) gene and capable of horizontal, interspecies transfer. No amplicons were yielded when primer pairs specific for the *int* gene, encoding the integrase protein that allows mobilization of Tn*916*, and for the intervening region between the *tet*(M) and *int* sequences, were used (data not shown). We did not find any of *tet*(W), *tet*(S), and *tet*(L) genes in this study.

Results of PCR screening for antibiotic resistance determinants in the *L. fermentum* strains were only partly consistent with those of phenotypic assays. Detection of *tet* genes in the strains HF-A1, HF-A4, HF-B1, 3-4, and 5-2 was in agreement with their resistance phenotypes. *L. fermentum* 5-1 sensitive to tetracycline was discovered to carry silent genes *tet*(K) and *tet*(M), and *L. fermentum* 3-4 sensitive to erythromycin was positive for *erm*(C). Detection of *erm*(B) in *L. fermentum* 5-1 was in agreement with its erythromycin resistant phenotype shown in the disc diffusion assay but was in contradiction with the results of MIC determination. These controversial results can be explained by dependence of susceptibility results on the method for antibiotic susceptibility assessment. It was shown that an increased inoculum size and an extended incubation time resulted in elevated antibiotic MICs for some species [14].

### 3.4. Transfer of Antibiotic Resistance Genes

We studied the transfer of tetracycline resistance genes by filter mating from six *L. fermentum* strains positive for *tet*(K) and *tet*(M) to a number of Gram-positive and Gram-negative bacteria—*Staphylococcus aureus*, *Staphylococcus epidermidis*, *Listeria monocytogenes*, *Acinetobacter baumannii*, *Citrobacter freundii*, and *Escherichia coli.* Also, *L. fermentum* 5-1 carrying the *erm*(B) gene and *L. fermentum* 3-4 carrying the *erm*(C) gene were used as donors, while *Staphylococcus haemolyticus* served as a recipient for erythromycin resistance genes. The recipient strains were chosen experimentally and met two criteria necessary for counterselection of transconjugants: (i) lack of resistance to erythromycin or tetracycline both in phenotypes and genotypes and (ii) presence of resistance to antibiotic active against tested *Lactobacillus* strains. Besides, these bacteria are members of the human gastrointestinal microbiota [40]. Coagulase-negative staphylococci *S. epidermidis* and *S. haemolyticus* usually colonize normal human skin but also may be isolated from the human gastrointestinal tract. Isolates from the intestinal tracts have been found to express virulence factors indicating their pathogenicity potentials [41].

The results showed that erythromycin and tetracycline resistance genes from tested *L. fermentum* strains cannot be transferred to the other bacteria used in this study via conjugation. No bacterial growth was observed in plates with the double selective medium, thus indicating that none of the recipient cells harbored erythromycin or tetracycline resistance (data not shown).

Lactobacilli and Gram-negative bacteria is an uncommon pair in studying the transferability of antibiotic resistance determinants. To date, there is only one report on conjugative transfer of antibiotic resistance genes from *L. fermentum* to other bacteria. The *erm*(B) gene from *L. fermentum* NWL24 was successfully transferred to *Enterococcus faecalis* 181 by filter mating [18]. However, it was demonstrated that gene transfer via conjugation can take place from Gram-positive to Gram-negative bacteria, and vice versa [42, 43]. The streptococcal conjugative transposon Tn*916* was shown to be able to cross the barrier between a variety of Gram-positive and Gram-negative bacteria, with subsequent expression in the new host [42]. Thus, absence of transconjugants in performed filter mating experiments coincides with the lack of Tn*916* in tested *L. fermentum* strains.

The disability to transfer genes for antibiotic resistance is considered an important parameter for the selection of the LAB strains for the food industry and probiotics [15]. This property is equally important for lactobacilli of normal human microbiota in order to avoid spread of resistance determinants among different species, including potential and obligate pathogens, in habitats with close association of densely packed microorganisms such as the intestine of humans. In all tested *L. fermentum* strains, *tet*(K) and *tet*(M) genes were shown to be located in plasmids and thus are likely to be acquired. Three *L. fermentum* strains (3-4, 5-1, and 5-2) isolated from dairy products represent a serious safety issue because of their tetracycline-resistant phenotype (strains 3-4 and 5-2) and simultaneous presence of tetracycline resistance genes *tet*(K) and *tet*(M) (all strains) along with erythromycin resistance genes *erm*(B) (strain 5-1) and *erm*(C) (strain 3-4). In this work, we have shown that all tested lactobacilli lack conjugative transposon Tn*916* and are not able to transfer tetracycline resistance genes to *S. aureus*, *S. epidermidis*, *L. monocytogenes*, *A. baumannii*, *C. freundii*, and *E. coli* by filter mating. Mating of *L. fermentum* 5-1 carrying the *erm*(B) gene and *L. fermentum* 3-4 carrying the *erm*(C) gene did not yield transconjugants with *S. haemolyticus* as well. These findings reduce the likelihood of the impact of tested lactobacilli to the spread of erythromycin and tetracycline resistance in the human microbiome and favor their safe status but still do not establish their complete safety. Our results highlight the need to include screening of antibiotic resistance into the current practices of safety evaluation mandatory before application of *Lactobacillus* strains as starter cultures or as probiotics.

## Figures and Tables

**Table 1:** Primers used in PCR amplifications.

Target gene	Primer sequence (5′-3′)	*T*_a_ (°C)	Amplicon size (bp)	Reference
*erm*(A)	F: AAGCGGTAAACCCCTCTGAG	52	441	[28]
	R: TCAAAGCCTGTCGGAATTGG			
*erm*(B)	ermB1-F: CATTTAACGACGAAACTGGC	60	425	[28]
	ermB1-R: GGAACATCTGTGGTATGGCG			
	ermB2-F: GAAAAGGTACTCAACCAAATA	59	639	[29]
	ermB2-R: AGTAACGGTACTTAAATTGTTTAC			
*erm*(C)	F: ATCTTTGAAATCGGCTCAGG	49	295	[28]
	R: CAAACCCGTATTCCACGATT			
*erm*(T)	F: TATTATTGAGATTGGTTCAGGG	55	395	[28]
	R: GGATGAAAGTATTCTCTAGGGATTT			
*mef*(A)	F: CTATGACAGCCTCAATGCG	52	1400	[30]
	R: ACCGATTCTATCAGCAAAG			
*Int*	F: GCGTGATTGTATCTCACT	50	1028	[31]
	R: GACGCTCCTGTTGCTTCT			
*Tet-int*	F: CGGATAGATAAAGTACGATA	52	2659	[32]
	R: TCACGTCTTTTTTCTGACAT			
*tet*(M)	tetM1-F: GAACTCGAACAAGAGGAAAGC	60	740	[32]
	tetM1-R: ATG GAAGCCCAGAAAGGAT			
	tetM2-F: GGTGAACATCATAGACACGC	58	401	[33]
	tetM2-R: CTTGTTCGAGTTCCAATGC			
*tet*(L)	tetL1-F: GTMGTTGCGCGCTATATTCC	55	696	[31]
	tetL1-R: GTGAAMGRWAGCCCACCTAA			
	tetL2-F: GTTTCGGGTCGGTAATTGGG	45	220	[28]
	tetL2-R: GCTATCATTCCACCAATCGC			
*tet*(K)	tetK1-F: TTATGGTGGTTGTAGCTAGAAA	55	348	[31]
	tetK1-R: AAAGGGTTAGAAACTCTTGAAA			
	tetK2-F: GTAGCGACAATAGGTAATAG	46	278	[28]
	tetK2-R: GCAACTTCTTCTTCAGAAAG			
*tet*(S)	F: GGAGTACAGTCACAAACTCG	55	335	[28]
	R: GGATATAAGGAGCAACTTTG			
*tet*(W)	F: GAGAGCCTGCTATATGCCAGC	55	168	[28]
	R: GGGCGTATCCACAATGTTAAC			
16S rRNA	27F: GAGTTTGATCCTGGCTCAG	51	1400	[34]
	1392R: ACGGTTACCTTGTTACGACTT			

**Table 2:** Characterization of *L. fermentum* strains with antibiotic resistance for erythromycin and tetracycline.

*L. fermentum strains*	Source	Erythromycin			Tetracycline			Phenotype^d^	Genotype^e^
		Zone of inhibition (mm)^a^	Characterization^b^	MIC^c^ (*μ*g/ml)	Zone of inhibition (mm)^a^	Characterization^b^	MIC^c^ (*μ*g/ml)		
HF-A1	Feces of healthy individuals	21.2 ± 2.9	S	0.25	15.7 ± 0.6	R	64	TET	*tet*(M)^p^, *tet*(K)^p^
HF-A4	Feces of healthy individuals	21.0 ± 1.4	S	0.25	15.5 ± 0.7	R	64	TET	*tet*(M)^p^, *tet*(K)^p^
HF-B1	Feces of healthy individuals	20.2 ± 3.4	MS	0.25	14.7 ± 0.6	R	64	TET	*tet*(M)^p^, *tet*(K)^p^
3-4	Sour-milk drink, “Tan,” “Chistaya Liniya,” Russia	21.0 ± 2.5	S	0.25	6.5 ± 2.1	R	16	TET	*erm*(C)^p^, *tet*(M)^p^, *tet*(K)^p^
5-1	Fermented baked milk, ryazhenka, “Chistoe pole,” Russia	6.5 ± 2.1	R	1	20.3 ± 1.4	MS	8	—	*erm*(B)^c^, *tet*(M)^p^, *tet*(K)^p^
5-2	Fermented baked milk, ryazhenka, “Nash product,” Russia	24.3 ± 2.5	S	0.25	14.5 ± 0.7	R	32	TET	*tet*(M)^p^, *tet*(K)^p^

^a^Diameters of the zones of inhibition determined by the disc diffusion method (mean value ± standard deviation) (Figure S1A). ^b^Based on standards mentioned in Materials and Methods, *L. fermentum* strains were characterized as either susceptible (S), moderately susceptible (MS), or resistant (R) to each antibiotic tested. ^c^The minimum inhibitory concentration (MIC) of antibiotics determined by the broth microdilution method as the lowest concentration of an antibiotic at which visible growth was inhibited. ^d^Based on MIC values, *L. fermentum* strains were characterized as erythromycin resistant (ERM) or tetracycline resistant (TET). ^e^Antibiotic resistance genes present in isolates of *L. fermentum* as detected using PCR: ^p^plasmid-located gene; ^c^chromosome-located gene (for details, see Figures S1B–S1D).

## Data Availability

The data used to support the findings of this study are included within the article.

## Supplementary Materials

Supplementary Materials Figure S1: phenotypic (A) and genotypic (B, C, and D) erythromycin and tetracycline resistance testing of *L. fermentum*. (A) Inhibition halos obtained by the agar disc diffusion method for *L. fermentum* 5-1 (1), *L. fermentum* HF-A1 (2), *L. fermentum* HF-A4 (3), *L. fermentum* HF-B1 (4), *L. fermentum* 3-4 (5), and *L. fermentum* 5-2 (6). PCR products of erythromycin (B) and tetracycline (C and D) resistance genes. (B) The PCR products amplified with *erm*(B)1 primer pair in plasmid DNA (lane 1) and total DNA (lane 2) of *L. fermentum* 5-1 and with *erm*(C) primer pair in plasmid DNA (lane 3) and total DNA (lane 4) of *L. fermentum* 3-4. The PCR products amplified with *tet*(K)2 (C) and *tet*(M)1 (D) primer pairs in plasmid DNA of *L. fermentum* HF-A1 (lane 1), *L. fermentum* HF-B1 (lane 2), *L. fermentum* HF-A4 (lane 3), *L. fermentum* 5-1 (lane 4), *L. fermentum* 3-4 (lane 5), and *L. fermentum* 5-2 (lane 6). Lane M, 1 kb DNA ladder.
