# Supplementary figures and images for: Characterization of Erythromycin and Tetracycline Resistance in Lactobacillus fermentum Strains
Source: Int J Microbiol. 2018 Nov 11;2018:3912326. doi: 10.1155/2018/3912326 (PMC6252201; doi:10.1155/2018/3912326)

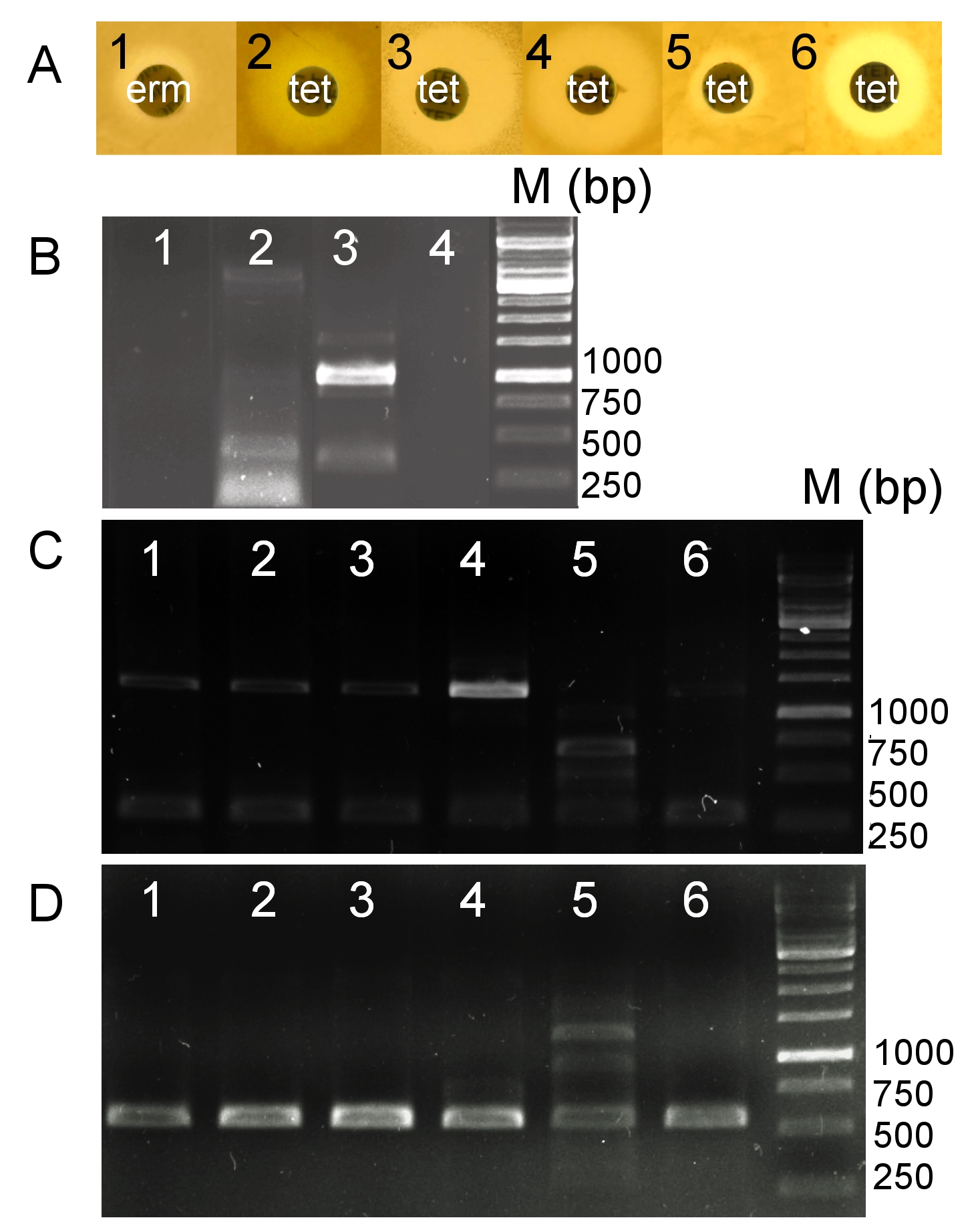

Supplement: Supplementary Materials — Figure S1: phenotypic (A) and genotypic (B, C, and D) erythromycin and tetracycline resistance testing of L. fermentum. (A) Inhibition halos obtained by the agar disc diffusion method for L. fermentum 5-1 (1), L. fermentum HF-A1 (2), L. fermentum HF-A4 (3), L. fermentum HF-B1 (4), L. fermentum 3-4 (5), and L. fermentum 5-2 (6). PCR products of erythromycin (B) and tetracycline (C and D) resistance genes. (B) The PCR products amplified with erm(B)1 primer pair in plasmid DNA (lane 1) and total DNA (lane 2) of L. fermentum 5-1 and with erm(C) primer pair in plasmid DNA (lane 3) and total DNA (lane 4) of L. fermentum 3-4. The PCR products amplified with tet(K)2 (C) and tet(M)1 (D) primer pairs in plasmid DNA of L. fermentum HF-A1 (lane 1), L. fermentum HF-B1 (lane 2), L. fermentum HF-A4 (lane 3), L. fermentum 5-1 (lane 4), L. fermentum 3-4 (lane 5), and L. fermentum 5-2 (lane 6). Lane M, 1 kb DNA ladder. [file 3912326.f1.jpg]
